# Evaluation of cholinergic enzymes and selected biochemical parameters in the serum of patients with a diagnosis of acute subarachnoid hemorrhage

**DOI:** 10.1515/tnsci-2022-0311

**Published:** 2023-10-18

**Authors:** Abdurrahman Aycan, Abdurrahim Tas, Asli Cilingir Yeltekin, Sama Amer Abbas El-Tekreti, Ayse Arslan, Mustafa Arslan, Nur Aycan

**Affiliations:** Department of Neurosurgery, Yuzuncu Yil University Faculty of Medicine, Van, Turkey; Department of Neurosurgery, Dicle University Faculty of Medicine, Diyarbakir, Turkey; Department of Chemistry, Yuzuncu Yil University Faculty of Science, Van, Turkey; Department of Nutrition and Dietetics, Yuzuncu Yil University Faculty of Health Sciences, Van, Turkey; Department of Pediatrics, Yuzuncu Yil University Faculty of Medicine, Van, Turkey

**Keywords:** subarachnoid hemorrhagic, GCS, brain-derived neurotrophic factor, tumor necrosis factor alpha, Caspase-3, acetylcholinesterase, butyrylcholinesterase

## Abstract

**Background:**

Spontaneous subarachnoid hemorrhage (SAH) is the most severe form of hemorrhagic stroke and accounts for 5–7% of all strokes. Several chemical enzymes and cytokines are thought to cause reactions that may affect the mortality and morbidity of SAH patients. This study aimed to examine the possible relationships between these parameters and the occurrence of SAH and the clinical–radiological parameters in patients with acute SAH.

**Methods:**

This study evaluated 44 patients, including 20 with SAH and 24 controls. We obtained blood from the patients and control groups, which was stored in heparinized tubes and used in determining tumor necrosis factor alpha (TNF-α), brain-derived neurotrophic factor (BDNF), acetylcholinesterase (AChE), caspase-3, and butyrylcholinesterase (BChE) enzymes.

**Results:**

TNF-α, BDNF, AChE, and BChE enzyme levels were not related to the Glasgow Coma scale (GCS) score in the patient group (*p* > 0.05), whereas higher enzyme levels of caspase-3 were associated with lower GCS scores (*p* < 0.05). The difference between the control and patient groups in terms of mean TNF-α levels was statistically significant (*p* < 0.01). The BDNF levels were statistically insignificant in the patient groups (*p* > 0.05). Caspase-3, AChE, and BChE levels were significantly different between the control and patient groups (*p* < 0.01).

**Conclusions:**

Our results may be valuable for predicting the prognosis, diagnosis, and follow-up of patients with SAH. However, further studies are required to elucidate the relationship between the clinical and radiological results in patients with SAH and certain enzymes, cytokines, and growth factors.

## 1 Introduction

Spontaneous subarachnoid hemorrhage (SAH) is a severe form of hemorrhagic stroke that constitutes 5–7% of fall strokes [1,2]. Additionally, 27% of stroke-related deaths are due to SAH [3]. Approximately 12% of patients die without medical attention, up to 33% die within 48 h, and only 40% of survivors return to their pre-bleeding functional status after treatment [4]. Ruptured brain aneurysms account for 85% of spontaneous SAHs [5]. Disability, memory loss, and poor language skills are common in patients with SAH [6]. These long-term outcomes result in a high socioeconomic burden [5]. Numerous factors affect the outcome, including the intensity of the initial hemorrhage, the degree of cerebral ischemia, and the patient’s age, as the most important [7]. Supportive treatments aim to prevent delayed cerebral ischemia and spasm in 30% of patients between 4 and 10 days [8]. However, no conclusive evidence has proven that these changes have improved morbidity and mortality despite improvements in the management and treatment of SAH-related complications over the last two decades [7].

Brain-derived neurotrophic factor (BDNF) is a growth factor that belongs to the neurotrophin family. It mediates synaptic plasticity, dendritic branching, inhibitory and excitatory neurotransmitter regulation, and neuronal growth [9,10]. BDNF is mainly retained in platelets and detectable in the serum of healthy persons [10]. Tumor necrosis factor alpha (TNF-α) is an immunomodulatory cytokine [11]. Atherosclerosis, a fundamental process in cerebral aneurysm formation, is crucial for vascular inflammation and dysfunction. Recent studies have shown that TNF-α is directly involved in the rupture and formation of cerebral aneurysms. These findings suggest that TNF-α inhibition is a promising therapeutic strategy [12]. Caspase-3 plays a critical role in the cell death pathway after acute brain injury in both experimental and clinical studies. Serum caspase-3 activity was associated with 30-day mortality after injury independent of other factors.

Additionally, studies have shown high caspase-3 plasma activity in the acute period and 6 months after stroke [13,14]. Acetylcholinesterase (AChE) and butyrylcholinesterase (BChE) break down acetylcholine. The main function of AChE is to break down acetylcholine in the synaptic gap and stop neurotransmission. However, it is found in small amounts in other body parts, and BChE is found in different concentrations in different brain parts [15,16].

To our knowledge, serum BDNF, TNF-α, caspase-3, AChE, and BChE levels have not been studied or evaluated in SAH patients. This study aimed to examine the possible relationships between the levels of these parameters and the occurrence of SAH and the clinical-radiological parameters in patients with acute SAH.

## 2 Materials and methods

In this study, 44 patients, 20 with SAH and 24 as controls, who underwent surgery in the Neurosurgery Clinic of Yüzüncü Yil University were evaluated. Between May 2021 and September 2022, the data of 64 patients who presented with spontaneous SAH due to vascular pathology in the Neurosurgery Clinic of Yüzüncü Yıl University were analyzed. Patients operated by the same surgical team were included in the study. Patients treated endovascularly and operated by different surgeons were excluded. The blood samples of 20 patients who met the exclusion criteria from 38 patients operated on by the same surgical team were included in the study. We obtained blood from the patient and control groups, stored it in heparinized tubes, and analyzed it in the laboratory. This study was conducted in compliance with the 2000 Declaration of Helsinki. This study was approved by the local Ethics Committee. Informed consent was obtained from all patients and their relatives included in the study. Neurological examinations and cranial computed tomography (CT), CT angiography, or digital subtraction angiography radiological examinations of patients brought to the Emergency Department of our center were performed. The Glasgow Coma scale (GCS) was used to assess neurological clinical status. The Fisher scale was used to grade SAH on CT. The control group comprised 24 healthy volunteers (11 females and 13 males) who were asymptomatic and underwent unremarkable medical and regular physical examinations. All control subjects were non-smokers and non-alcoholic.

### 2.1 Exclusion criteria

Exclusion criteria included alcohol abuse, abuse of intravenous drugs, pregnancy, use of antioxidant supplements, and chronic diseases such as hypertension, diabetes mellitus, liver or kidney diseases, rheumatoid arthritis, and pulmonary and coronary artery diseases.

### 2.2 Blood samples

The obtained blood was centrifuged at 3,500 rpm for 10 min, and the upper plasma was separated for further analysis. Plasma samples were frozen at −18°C, and a survey of the study was conducted.

### 2.3 AChE/BChE enzyme activity

The AChE and BChE enzymes were spectrophotometrically determined according to the Ellman method. The thiol ester acetylthiocholine was used instead of oxy ester acetylcholine as the substrate in the Ellman method. Acetylthiocholine is hydrolyzed by AChE according to the Ellman method, and the thiocholine released as a result of hydrolysis is combined with the Ellman reagent 5,5′-dithio-bis-(2-nitrobenzoic acid) (DTNB) The yellow-colored chromophore 5-thio-2-nitrobenzoic acid (TNB) was formed during the reaction. The formation rate (intensity of color) of this yellow compound at the end of the reaction was determined by measuring the absorbance at 412 nm [17]. The intensity of the yellow color was directly proportional to the AChE/BChE enzyme activity.

### 2.4 Determination of apoptosis: the Caspase-3 level

**Figure 1:**
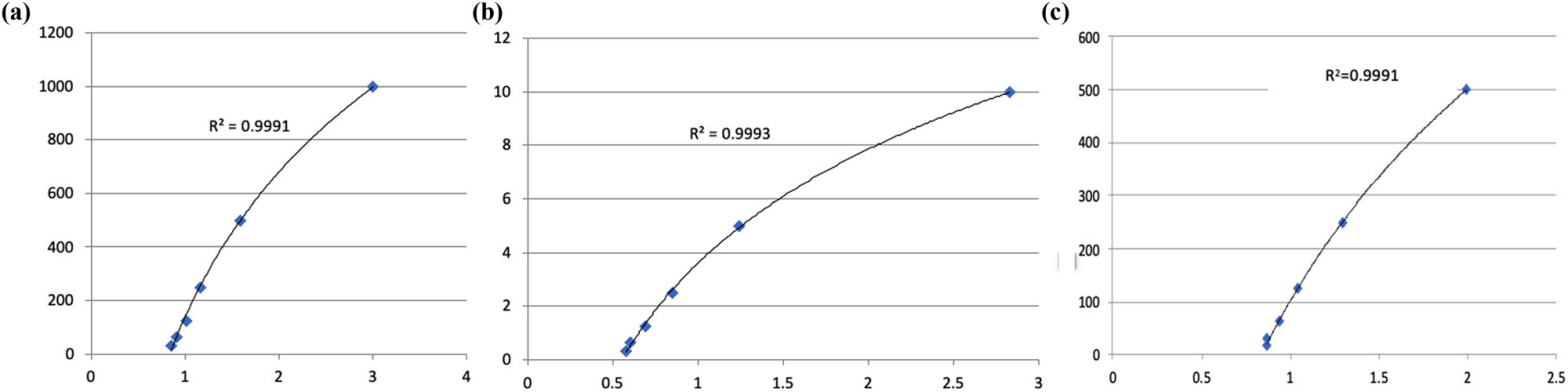
(a) Caspase-3 (ng/ml) standard graph for the ELISA method, (b) BDNF (pg/mL) standard graph for the ELISA method, and (c) TNF-α (pg/ml) standard graph for ELISA method.

Caspase-3 enzyme activity in the control and experimental group samples was determined using the “Caspase-3 Analysis Kit” (Fish [CASP3] enzyme-linked immunosorbent assay [ELISA] Kit [Catalog No: 201-00-0031] [SunRed]). The important step in this analysis was the determination of the product formed by the reaction of the substrate with the caspase-3 enzyme. Readings were performed in 10 min ELISA (plate reader) devices at 450 nm absorbance. The caspase-3 levels in the samples were calculated according to the formula created by drawing a standard graph suitable for the optical densities and concentrations of caspase-3 standards (Figure 1).

### 2.5 BDNF

**Figure 2:**
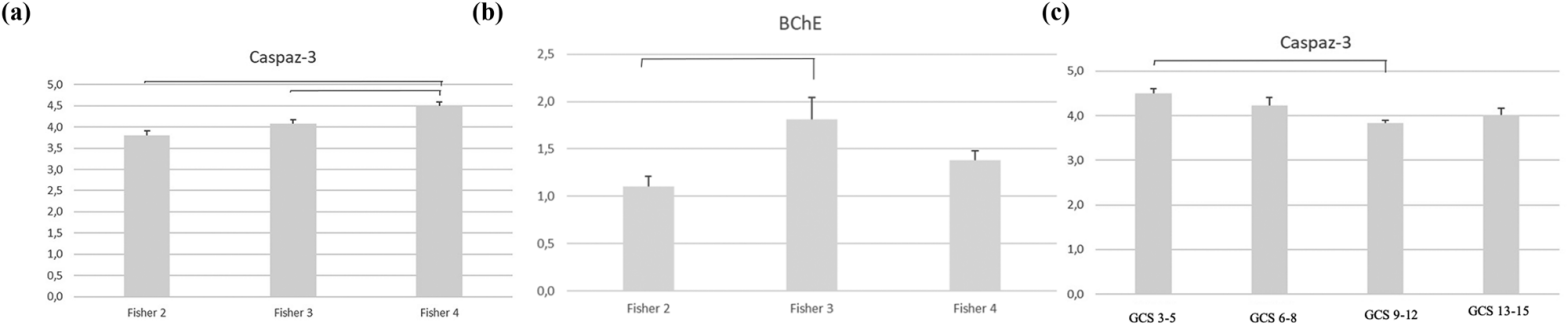
(a) Comparison of Caspase-3 values in Fisher Groups. (b) Comparison of BChE values in Fisher Groups. (c) Comparison of Caspase-3 values in GKS Groups.

Serum BDNF levels were determined using a BDNF ELISA kit. The standards were prepared by bringing the kit materials to room temperature half an hour before starting the study and were added to the MicroELISA Strip Plate. We then re-applied, the necessary kit procedures, and the measurements were performed within 10 min in the ELISA. The level of IL-6 was evaluated using a specific ELISA kit according to the provider’s guide (E0026Fi, BT LAB) (Plate Reader) device with an absorbance of 450 nm. The BDNF levels in the samples were calculated according to the formula created by drawing a standard graph suitable for the optical densities and concentrations of BDNF standards (Figure 2).

### 2.6 TNF-α levels

**Figure 3:**
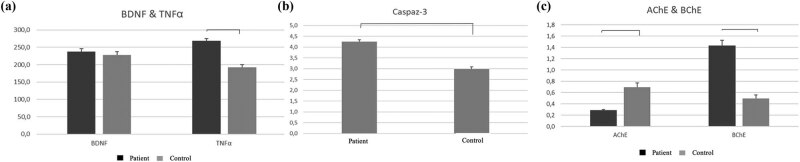
(a) Comparison of BDNF and TNF-α values in patient and control Groups. (b) Comparison of Caspase-3 values in patient and control Groups. (c) Comparison of AChE and BChE values in patient and control Groups.

TNF-α levels in samples were measured by ELISA using commercial kits (Human HIF-1α ELISA kit, Human VEGF ELISA kit, Human TNF-α ELISA kit, Catalog No: 201-00-0041, Sunred Biotechnology, Shanghai, China) following the manufacturer’s guidelines. Readings were taken in 10 min ELISA (plate reader) devices at 450 nm absorbance. TNF-α levels in the samples were calculated according to the formula created by drawing a standard graph suitable for the optical densities and concentrations of TNF-α standards (Figure 3).

### 2.7 Statistical analysis

The results are expressed as mean ± standard deviation. Parametric variables were compared using the *t*-test. Qualitative variables were assessed using the chi-square test. The results were considered statistically significant at a *p*-value of 0.05. Data were analyzed using Statistical Package for the Social Sciences for Windows Version 20.0.

The standard graphs of the Eliza methods are plotted as shown in Figure 1(a–c).


**Ethical approval:** The research related to human use complied with all the relevant national regulations, institutional policies, and is in accordance with the tenets of the Helsinki Declaration, and has been approved by the author’s institutional review board or equivalent committee.
**Informed consent:** Informed consent has been obtained from all individuals included in this study.

## 3 Results

This prospective study included 30 patients with acute SAH due to aneurysms (17 females, 13 males) and 24 healthy volunteers (11 females, 13 males). The GCS scores at the time of admission were 13–15, 9–12, 6–8, and 3–5 [3,6,7,14]. Moreover, a single aneurysm was detected in 19 patients, and multiple aneurysms were detected in 11 patients (Table 1). Serum BDNF, TNF-α, caspase-3, AChE, and BChE levels were measured in 20 patients with spontaneous SAH due to aneurysms (Table 2).

**Table 1:** Evaluation of the demographic characteristics of the groups

		Patients (*n* = 30)	Control
Age (mean ± SE)		55.8 ± 2.93	53.6 ± 2.27
Gender	Female	17	11
	Male	13	13
Fisher score (mean ± SE)		3.36 ± 0.14	—
GKS at the time of admission to the hospital	13–15	3	—
	9–12	6	
	6–8	7	
	3–5	14	
Aneurysm number	Single	19	—
	Multiple	11	
Comorbidities	Diabetes mellitus	2	—
	Hypertension	12	—
	Inflammatory disease	—	—

**Table 2:** Comparison of the demographic characteristics and biochemical parameters of the groups

Enzyme	Groups	*N*	Mean	SE	*t*-Test		
					*t*	SD	*p*
TNF- α	Patients	20	268.64	9.21	6.29	39	0.00012
	Control	24	192.28	7.89			
BDNF	Patients	20	237.42	11.49	0.63	39	0.533
	Control	24	227.82	10.01			
Caspase-3	Patients	20	4.25	0.1	8.84	41	0.00022
	Control	24	2.98	0.1			
AChE	Patients	20	0.29	0.01	−5.36	21	0.0002
	Control	20	0.69	0.07			
BChE	Patients	20	1.43	0.12	6.91	28	0.0004
	Control	20	0.49	0.06			

*N*: number, statistical significance (*p* < 0.05).

The analysis of mean TNF-α, BDNF, caspase-3, AChE, and BChE enzyme levels in the patient group revealed no statistical significance (*p* > 0.05). The mean TNF-α, BDNF, and AChE enzyme levels in the patient group according to Fisher scoring were not statistically significant (*p* > 0.05). The examination of the results of the caspase-3 enzyme in terms of Fisher scoring in the patient group revealed Fisher scores between 3 (4.07 ± 0.1) and 4 (4.48 ± 0.1; *p* < 0.05) and between 2 (3.80 ± 0.1) and 4 (4.48 ± 0.1; *p* < 0.05), with a statistically significant difference. The comparison of the mean BChE enzyme revealed statistical significance between those with Fisher scores of 2 (1.1 ± 0.11) and 3 (1.81 ± 0.23; *p* < 0.05).

**Figure 4:**
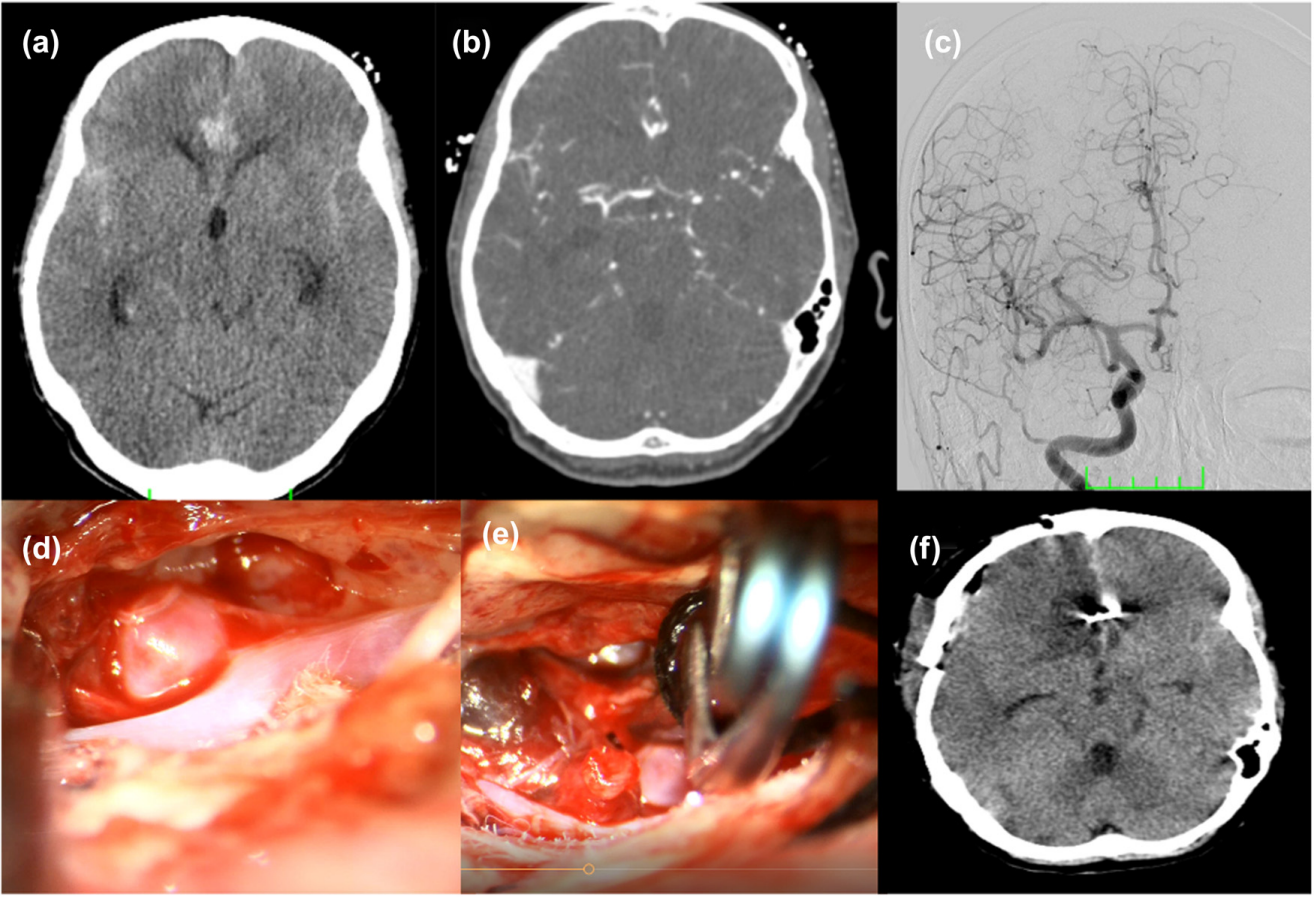
Representative images (a) Preoperative cranial CT: Fisher grade 2, (b) Preoperative Cranial CT angiography, (c) Preoperative DSA, (d) Intraoperative Acom Aneurysm, (e) Peroperative aneurysm clips, (f) Postoperative cranial CT.

The analysis of the mean levels of TNF-α, BDNF, AChE, and BChE in the patient group in terms of GCS revealed no statistical significance (*p* > 0.05). The comparison of the mean caspase-3 enzyme revealed statistical significance in patients with GCS of 3–5 (4.5 ± 0.1) and 9–12 (3.83 ± 0.65; *p* < 0.05). A significant inverse relationship was found between the GCS and Fisher scores, with Spearman’s correlation coefficient (*r* = 0.55; *p* = 0.001).

The mean TNF levels were significantly different between the control and patient groups, with higher mean TNF values in the patient group (*p* < 0.01). The mean BDNF values of the patient group were higher (*p* > 0.05), although the difference between the control and patient groups in terms of mean BDNF levels was not statistically significant.

The difference in the mean caspase levels between the control and patient groups was statistically significant (*p* < 0.01). The mean caspase value in the patient group was significantly higher (*p* < 0.01).

The mean AChE levels were significantly different between the control and patient groups, with higher mean AChE values in the control group (*p* < 0.01). The mean BChE levels were significantly different between the control and patient groups, with higher mean BChE values in the patient group (*p* < 0.01). Preoperative CT, CT Angiography, DSA Angiography, Intraoperative and postoperative CT images of a patient with SAH due to aneurysm are shown in Figure 4.

## 4 Discussion

Subarachnoid hemorrhage is the displacement of blood in the brain into the subarachnoid space for various reasons. The most common cause of SAH is an aneurysm [5].

SAH, which accounts for 5–7% of all strokes, is the most severe form of hemorrhagic stroke. It is essential to diagnose this disease with high mortality and morbidity and to start treatment as soon as possible [18].

High-quality, unenhanced, high-resolution CT performed within the first 48 h of SAH confirmed the presence of SAH in 95% of cases. Lumbar puncture is the most sensitive test for subarachnoid hemorrhage (SAH). However, this may yield false-positive results. Cerebral angiography is the “gold standard” method for evaluating cerebral aneurysms [19,20].

Intraventricular hemorrhage (13–28%), intraparenchymal hemorrhage (more common in distal aneurysms), and rarely subdural hemorrhage (2–8%) patterns can be seen with aneurysmal subarachnoid hemorrhage. Intraventricular bleeding is most commonly observed in ACom A aneurysms. This is thought to be because of its close proximity to the ventricle [21].

The serum concentrations of BDNF, TNF-α, caspase-3, AChE, and BChE in patients with spontaneous SAH were investigated. To our knowledge, this is the first study to examine the serum levels of BDNF, TNF-α, caspase-3, AChE, and BChE in patients with spontaneous SAH. Several chemical enzymes, factors, and cytokines are thought to cause reactions that may affect the mortality and morbidity of SAH patients. Therefore, changes in the serum levels of these patients are important. The mean BDNF, TNF-α, caspase-3, as well as AChE and BChE values of the patient group with spontaneous SAH were higher than those of the controls.

Mitochondrial overproduction and hemoglobin autooxidation have a poor prognosis, although various research sources lead to excessive free radical production [22]. The leakage of superoxide anions from the mitochondria is attributed to ischemic consequences and the role of free radicals in early brain damage after SAH is very high [23]. Ischemia after SAH causes calcium accumulation in the mitochondria [24]. Hence, it disrupts the mitochondrial membrane potential and increases permeability [25]. Membrane potential rebuilding is accomplished by consuming oxygen, which leads to superoxide formation [23]. Excess free radicals cause oxidative stress, which in turn causes lipid, protein, and DNA damage [23,24]. This damage leads to increased free radicals in the plasma of patients with SAH, a depleted antioxidant system, oxidative stress, and eventually neuronal death.

BDNF is widely used as a marker for the healing and regeneration of damaged neurons. Several animal studies have revealed the role of BDNF in neurogenesis, angiogenesis, brain repair, and synaptic plasticity. Therefore, BDNF is considered to be an essential factor [10,26,27]. Karatanli et al. measured BDNF levels in the plasma of stroke patients and compared them with those of a control group, revealing high BDNF levels in stroke patients. However, they did not find a relationship between BDNF levels and the infarct volume. This highlights the potential role of BDNF, measured within the acute stage of stroke (after 3 weeks), as an indicator of stroke results [28]. However, this difference was not significant during the acute phase of the study. Di Lazzaro et al. reported solidness of serum BDNF in the acute stroke stage in ten patients with first-time acute ischemic stroke [29].

Most cerebrovascular diseases are associated with atherosclerosis, an inflammatory disease in which inflammatory mediators such as several cytokines may be involved in atheroma formation. TNF-α is a potent immunomodulator and proinflammatory cytokine that plays a role in many pathological processes such as atherosclerosis [30]. Llamas Sillero et al. evaluated 308 patients and found a high prevalence of cerebrovascular disease among those with TNF-α polymorphisms. These results were expected because atherosclerosis is the leading cause of cerebrovascular diseases [31]. Dihydrothalidomide, which inhibits TNF-α synthesis, regressed the arterial wall changes in aneurysms induced by hemodynamic stress and hypertension in mice. Another animal study revealed less common aneurysm development and rupture of developing aneurysms in TNF-α knockout mice.

Additionally, TNF-α levels increased more in ruptured aneurysms than in non-ruptured ones [32]. Our study revealed significantly higher TNF-α levels in patients with SAH. Activation of caspases plays a crucial role in apoptotic events in acute and persistent neurological disorders such as stroke, TBI, and other neurodegenerative infections [33,34,35].

The available published information suggests that apoptotic cell death after brain injury and neurodegeneration is fundamentally related to caspase-3 activation, although information on the association of caspase-7 is restricted [13]. This information expands on caspase-3 activity and neuronal apoptosis within the perivascular region, suggesting that caspase-3 is involved in cerebrovascular injuries. Previous studies have revealed that caspase-3 is increased in traumatic brain injury, stroke, Alzheimer’s disease, intracerebral hematoma, and aneurysmal and traumatic sacs [36,37,38,39,40]. Additionally, a study involving patients with intracerebral hemorrhage in the basal ganglia without surgical hematoma evacuation revealed an association between serum caspase-3 levels and late mortality (at 6 months) [41]. Our study revealed a significantly increased caspase-3 level compared with that in the control group.

AChE and BChE are enzymes that break down acetylcholine in the body. AChE breaks down acetylcholine in the synaptic gap and stops neurotransmission. However, it is also found in small amounts in other body parts [15]. BChE is expressed primarily in glial cells, especially astrocytes, during differentiation into AChe found in neurons within the human brain. However, BChE is also found in neurons primarily localized within the amygdala, hippocampus, and thalamus [16,42]. Low blood AChE and cholinergic state activity increase cardiac and ischemic stroke mortality, thereby increasing the risk of major adverse cardiovascular events. The BChE increased during the post-ischemic stroke period. A severe decrease in AChE activity after stroke has been associated with a poor prognosis [43]. Consistent with the literature, AChe values were significantly lower in the patient group than in the control group. In contrast, the BChe values were significantly higher in the patient group in our study.

The cross-sectional nature of our study is a limitation. Another factor is the inability to measure the levels of relevant parameters studied in patients in the short and medium term after treatment, which limits our study. Finally, the number of patients with SAH included in this study was relatively small. Hence, the relevant parameters studied in patients will increase the power to detect differences in a larger number of patients.

## 5 Conclusion

Therefore, TNF-α, BDNF, AChE, and caspase 3 values in the serum of patients with SAH were higher in the patient group, and BChE values were lower in the patient group than in the control group. Additionally, the relationship between the relevant parameters and GCS and Fisher score was examined, and the relationship between the neurological picture and radiological findings was confirmed. These findings may have prognostic and diagnostic value in patients with SAH. Further studies are needed to elucidate the relationship between clinical and radiological findings in patients with SAH and certain enzymes, cytokines, and growth factors.

## Abbreviations

AChE: acetylcholinesterase
BChE: butyrylcholinesterase
BDNF: brain-derived neurotrophic factor
CT: computed tomography
ELISA: enzyme-linked immunosorbent assay
GCS: Glasgow coma scale
SAH: subarachnoid hemorrhage
TNF-α: tumor necrosis factor
